# Enzymatic Characterization of Fructose 1,6-Bisphosphatase II from *Francisella tularensis*, an Essential Enzyme for Pathogenesis

**DOI:** 10.1007/s12010-017-2512-6

**Published:** 2017-05-25

**Authors:** Hiten J. Gutka, Nina M. Wolf, Jasper Marc G. Bondoc, Farahnaz Movahedzadeh

**Affiliations:** 1 Institute for Tuberculosis Research, College of Pharmacy, University of Illinois at Chicago, Chicago, IL USA; 2 Department of Medicinal Chemistry and Pharmacognosy, College of Pharmacy, University of Illinois at Chicago, Chicago, IL USA; 3 Present Address: Oncobiologics Inc., Cranbury, NJ USA

**Keywords:** Gluconeogenesis, *Francisella tularensis*, Fructose-1,6-bisphosphatase, Enzymatic activity, *glpX*

## Abstract

The *glpX* gene from *Francisella tularensis* encodes for the class II fructose 1,6-bisphosphatase (FBPaseII) enzyme. The *glpX* gene has been verified to be essential in *F. tularensis*, and the inactivation of this gene leads to impaired bacterial growth on gluconeogenic substrates. In the present work, we have complemented a *∆glpX* mutant of *Escherichia coli* with the *glpX* gene of *F. tularensis* (FTF1631c). Our complementation work independently verifies that the *glpX* gene (FTF1631c) in *F. tularensis* is indeed an FBPase and supports the growth of the *ΔglpX E. coli* mutant on glycerol-containing media. We have performed heterologous expression and purification of the *glpX* encoded FBPaseII in *F. tularensis*. We have confirmed the function of *glpX* as an FBPase and optimized the conditions for enzymatic activity. Mn^2+^ was found to be an absolute requirement for activity, with no other metal substitutions rendering the enzyme active. The kinetic parameters for this enzyme were found as follows: K_m_ 11 μM, V_max_ 2.0 units/mg, k_cat_ 1.2 s^−1^, k_cat_/K_m_ 120 mM^−1^ s^−1^, and a specific activity of 2.0 units/mg. Size exclusion data suggested an abundance of a tetrameric species in solution. Our findings on the enzyme’s properties will facilitate the initial stages of a structure-based drug design program targeting this essential gene of *F. tularensis*.

## Introduction

*Francisella tularensis* (*F. tularensis*) is the etiologic agent of tularemia. This highly virulent bacterium is considered high risk for use as a biological weapon since the minimal infectious dose for human is only 10 bacteria [1]. Identifying new drugs and antibiotics against such pathogens is vital. Therefore, the execution of a rational structure-based drug design study targeting key regulatory proteins and enzymes in *F. tularensis* would ultimately lead to a novel antibiotic which could potentially become a drug.

The virulence of several *F. tularensis* SCHU S4 strain mutants was assessed by following the outcome of infection after intradermal infection. In this study, the virulence of 20 in-frame deletion mutants and 37 transposon mutants were assessed [2]. The majority of the mutants did not show an increase in prolonged time to death. However, mutations in six unique targets resulted in significantly prolonged time to death and mutations in nine targets, including *glpX*, and led to marked attenuation with an LD_50_ of >10^3^ CFU. Compared to the wild-type strain with the LD_50_ of one CFU, *glpX* mutant showed a marked attenuation with an LD_50_ of 10^7^ CFU or greater [2]. The extreme attenuation of the *∆glpX* mutant suggests that *glpX* is required for the virulence of *F. tularensis* in vivo [2].

More recently, the *glpX* of *F. tularensis* was demonstrated to be an essential component for bacterial growth and a requirement for mouse infection [3]. Growth of wild-type and the *∆glpX* mutant of *F. tularensis* were unaffected by glucose and ribose, while the *∆glpX* mutant greatly impaired growth on glycerol, pyruvate, and an amino acid cocktail. A challenge of wild-type and *∆glpX* mutant *F. tularensis*, subspecies *F. novicida*, in mice resulted in an 80% survival rate for mice infected with the *∆glpX* mutant after 10 days, while mice who received the wild-type bacteria all died after 3 days [3].

Our work on the *glpX* encoded FBPase II in *Mycobacterium tuberculosis* indicates that it is essential for the long-term survival and proliferation in mice model [4]. We were able to successfully demonstrate that the *glpX* encoded FBPase is an FBPase II [5]. Further, we reported the expression, purification and biochemical characterization of this enzyme in *M. tuberculosis* [6, 7]. The sequence similarity of the *glpX*-encoded proteins in both *M. tuberculosis* and *F. tularensis* promoted us to investigate the properties of this protein in *F. tularensis* as well.

There are five known classes of FBPases [8–10]. Most organisms contain a mixture of Class I and II. Class I FBPases, the most common form, are found in several eukaryotes and some prokaryotes while Class II is mainly found in bacteria with a few cases in eukaryotes [11]. Class III was defined for a unique FPBase from *Bacillus substilus*, structurally unrelated to the other classes [11]. Class IV from archaea have both inositol monophosphatase and fructose-1,6-bisphosphatase activity [12]. Class V FBPases are found in thermophile prokaryotes and contain an unusual 4-layer α-β-β-α fold instead of the common 5-layer α-β-α-β-α fold [13]. *Escherichia coli* has both Class I (*fbp*) and Class II (*glpX*). To date, *F. tularensis* contains one proposed FBPase Class II, *glpX*. The *glpX* gene is required for the virulence of *F. tularensis* in vivo [2, 3]. There is no known Class I fructose 1,6-bisphosphatase (FBPase) in *F. tularensis*, and it is expected that the *glpX* gene product is responsible for the catalysis of fructose 1,6-bisphosphate (F16BP) due to sequence similarity. However, the exact biochemical activity, or function of the *glpX* gene product, fructose 1,6-bisphosphatase (FBPaseII), has not been experimentally verified. Here, we have biochemically characterized the FBPaseII of *F. tularensis* (*Ft*FBPaseII) to verify functionality, specificity and stability. We also describe preliminary structural characterization by size exclusion chromatography of *Ft*FBPaseII. These experiments serve as a foundation for a structure-based drug design strategy targeting the *Ft*FBPaseII enzyme.

## Materials and Methods

All materials were purchased from Thermo Fisher Scientific, Waltham, MA, unless noted. Enzymes for the secondary assay and substrate compounds were purchased from Sigma Aldrich, St. Louis, MO.

### Cloning

The *F. tularensis glpX* gene was codon optimized, synthesized, and cloned into a pET15b vector suitable for genetic complementation studies and recombinant expression (CelTek Bioscience, Franklin, TN). The construct was sequenced for its accuracy, subjected to restriction digestion with BamHI/XbaI, and the insertion of the *FtglpX* gene was confirmed.

### Complementation

The pET15b-*FtglpX* construct was transformed into *E. coli* strain JLD2402 (TL524 *glpX*::Spc^r^ Δ*fbp287 zjg920*::Tn*10*), which lacks both *fbp* and *glpX,* (JLD2402*-*pET15b-*FtglpX*), and JLD2404 (TL524 *glpX* ^*+*^ Δ*fbp287 zjg920*::Tn*10*), which lacks only *fbp* (JLD2404*-*pET15b-*FtglpX*) [14]. Antibiotic-resistant transformants were grown on minimal media plates containing glucose or glycerol as the sole carbon source and isopropyl β-d-1-thioglactopyranoside (IPTG) to induce expression of the *glpX* gene. Additionally, the pET15b-*FtglpX* construct was transformed into *E. coli* strain JB108 (BL21(DE3) *zjg920*::Tn*10* ∆*fbp287*) (JB108::pET15b-*FtglpX*) [14], which was grown in minimal medium containing glycerol. Appropriate control strains of *E. coli* BL21(DE3) (Novagen, Billerica, MA), untransformed JB108, and JB108 strain transformed with pET15b (JB108::pET15b) were also run with appropriate concentrations of IPTG. The plates were incubated at 27 °C for 36 h to visually confirm bacterial growth.

### Species Primary Sequence Comparison

The *glpX* sequence of *F. tularensis* was obtained from the KEGG database (FTF1631c), the *E. coli* sequence was obtained from SWISSPROT (accession no. P28860), the *C. glutamicum* sequence was obtained from GenBank (accession no. 19552240), the *M. tuberculosis* sequence was obtained from the Tuberculist server (http://genolist.pasteur.fr/TubercuList/; gene name Rv1099c), and the *M. smegmatis* sequence was derived from genomic sequences obtained from the Institute for Genomic Research website (http://www.tigr.org/). The exact start residue of the proteins is known only for *C. glutamicum* [15].

### Expression and Purification

The pET-15b-*FtglpX* construct expressing N-terminal histidine-tagged *Ft*FBPaseII was transformed into *E. coli* strain BL21(DE3). The positive colonies were grown on LB agar containing ampicillin (100 μg/mL). Then, the transformants were grown overnight in LB broth (with ampicillin at 100 μg/mL) at 37 °C in an incubator shaking at 180 rpm. After 16 h, 1.0 mL of this culture was transferred to 100 mL of LB broth (with ampicillin at 100 μg/mL) and incubated at 37 °C. When the OD_600_ reached 0.6, the culture was induced with IPTG (1 mM). After 5 h, the cell pellet was harvested and frozen at −20 or −80 °C until further use.

Purification of the *Ft*FBPaseII was performed in a similar manner as described for *Mt*FBPaseII [6], except that the final buffer exchange and concentration of the purified protein was performed using an Amicon-15 Ultracel 30 K centrifuge concentrator or Zeba spin desalting column. Protein molecular mass was determined using electron spray ionization mass spectrometry coupled with HPLC (Thermo Orbitrap Velos Pro MS, Agilent 1200 nano HPLC) through the Research Resources Center at UIC.

### Size Exclusion Chromatography

Size exclusion chromatography (SEC) was performed on an ÄKTA purifier FPLC system in a similar manner to that described for *Mt*FBPase [6]. Retention times were determined by monitoring the absorbance at 280 nm. *Ft*FBPaseII was injected at a concentration of 1.0 mg/mL. Total protein quantification was performed using the Pierce 660-nm kit. Bovine serum albumin (BSA) was used as the standard [ThermoFisher Scientific]. The relative elution (*K*_*av*_) and hence the molecular weight for *F. tularensis* FBPaseII was estimated as described for *Mt*FBPaseII using thyroglobulin (bovine) 670 kDa, gamma globulin (bovine) 158 kDa, ovalbumin (chicken) 44 kDa, myoglobulin (horse) 17 kDa, and vitamin B12 1350 Da. Since raw data was no longer available, previously acquired images were uploaded to http://arohatgi.info/WebPlotDigitizer and the data was processed with the Δx step interpolation algorithm of 0.01 units and 0% smoothing to create Fig. 4a, b (WebPlotDigitizer, Austin, TX).

### Enzymatic Activity

Assays were conducted in 50 mM Tris pH 8.0 with 100 mM Mn^2+^, 50 nM *Ft*FBPaseII, and 100 μM F16BP unless otherwise indicated. Protein concentration was determined by absorbance at 280 nm with extinction coefficient 12,950 M^−1^ cm^−1^. Assays were performed at room temperature, approximately 22–24 °C.

A malachite green assay [16] was used to test for metal requirements, buffer system, and substrate specificity. Enzymatic solutions were allowed to react for 15 min before being quenched with the malachite green agent. The absorbance at 630 nM was measured after an additional 5 min for dye development. The following metals, as chloride salts at 100 mM, were tested for absolute requirement for activity: Mg^2+^, Ca^2+^, Zn^2+^, Fe^2+^, Cu^2+^, Co^2+^, Ni^2+^, K^+^, Na^+^, and Mn^2+^. Different buffering systems were tested at pH 7.5 and 50 mM buffer component. The pH of 50 mM Tris was screened (7.0–9.0 pH units). The amount of phosphate released was determined by using a calibration curve of potassium phosphate standard in the presence of 50 mM Mn^2+^. Different substrates at 1 mM (d-fructose-1,6-bisphosphate, Sn-glycerol-6-phosphate, 3-phosphoglycerate, d-mannose-6-phosphate, d-fructose-6-phosphate, d-glucose-6-phosphate, d-fructose-1-phosphate, d-ribulose-1,5-bisphosphate, d-glucose-1,6-bisphosphate) were tested for substrate specificity against *Ft*FBPaseII and adjusted for autophosphohydrolysis.

A coupled assay [6, 15], measuring production of NADPH at 340 nm, was used to determine enzymatic parameters and analyzed with a non-linear fit of Michaelis-Menten and k_cat_ equations (fixing E_t_ at 50 nM) using GraphPad Prism version 7.0b for Mac (GraphPad Software, La Jolla, CA). Using the coupled assay, the following optimal conditions were found where *Ft*FBPaseII is the limiting reagent: 5 units/mL of phosphoglucoisomerase, 2 units/mL of glucose-6-phosphate dehydrogenase, 0.3 mM NADP^+^, 15 μM F16BP, and 50 nM FBPaseII. Li^+^ sensitivity was assessed with concentrations up to 100 mM. The reaction was monitored for 3.5 min. Enzyme inhibition by adenosine diphosphate (0.6–4 mM) and free phosphate (0.2–10 mM) was calculated relative to the unaccompanied enzyme. The residual activity after heating was assessed by incubating protein samples for 30 min at various temperatures in a water bath (10–80 °C), returning to ice for 15 min and assaying against a sample on ice for the same length of time.

## Results

### *F. tularensis glpX* Encodes a Class II FBPase which Functionally Complements an *E. coli ∆glpX/∆fbp* Strain

In case of *E. coli*, it has been proven that the *glpX*-encoded FBPase II is not crucial for cell growth and proliferation since a strain lacking the *glpX* gene (JLD2403 (fbp1 glpX::Spcr)), successfully grows on LB or glucose, fructose, succinate, or glycerol minimal medium, in both aerobic and anaerobic conditions when the *fbp*-encoded FBPase I (major FBPase in *E. coli*) is present.

**Fig. 1:**
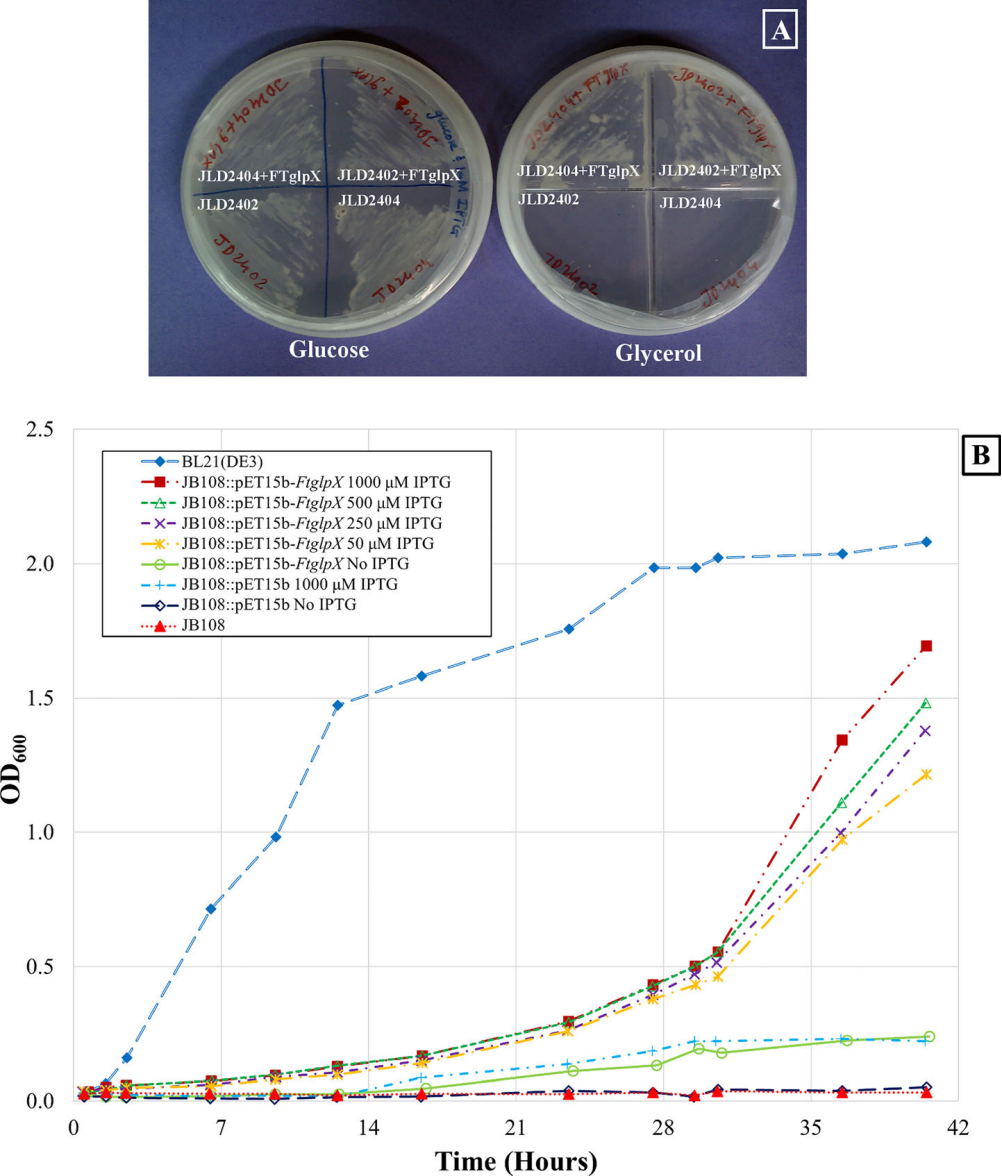
Growth pattern of the glucose-dependent *E. coli* strains transformed with *FtglpX*. JLD2402 and JLD2404 *E. coli* strains transformed with the pET15b-*FtglpX* construct were streaked onto minimal agar containing glucose (left) or glycerol (right) as the carbon source (**a**). The transformed strains grew on minimal agar with glycerol but not the control (untransformed) strains. *E. coli* strains JB108, JB108::pET15b, JB108::pET15b-*FtglpX* in which *Ft*FBPaseII is expressed from the T7 promoter and BL21(DE3) (*fbp* ^*+*^ *glpX* ^*+*^) control strain were grown in minimal medium containing glycerol. The growth of JB108::pET15b-*FtglpX* increased in an IPTG concentration (0 to 1000 μM)-dependent manner (**b**). The starter culture was grown overnight in minimal medium containing glucose. The IPTG levels utilized for strain JB108::pET15b-*FtglpX* during growth were 0, 50, 250, 500, and 1000 μM. No IPTG was used for each of BL21(DE3) (*fbp* ^*+*^ *glpX* ^*+*^), JB108, JB108::pET15b, and JB108::pET15b-*FtglpX* strains, which served as controls. This experiment was repeated twice. Samples for measuring FBPase activity were withdrawn at 24 h post-IPTG induction; the cultures were also supplemented with ampicillin (100 μg/mL)

*E. coli* strains lacking the FBPase I grow normally on glucose but are unable to grow on minimal medium supplemented with glycerol or other gluconeogenic substrates, indicating that chromosomal *glpX* even if present does not fully compensate the loss of *fbp* expression. The pET15b-*FtglpX* construct was introduced into *E. coli* strains JLD2402 (TL524 glpX::Spcr Δfbp287 zjg-920::Tn10) and JLD2404 (TL524 glpX1 Δfbp287 zjg-920::Tn10), the ampicillin resistant colonies were streaked on minimal media plates containing either glucose or glycerol as the sole carbon source, and IPTG was used to induce the expression of the *glpX* gene. While the control strains (untransformed JLD2402 and JLD2404) only grew on glucose as a sole carbon source, the pET15b-*FtglpX* construct transformed into JLD2402 and JLD2404 grew on either glucose or glycerol as the sole carbon source (Fig. 1a). This genetic complementation is indicative of FBPase activity of the *F. tularensis glpX* gene product. However, it is noteworthy that such complementation occurs at 27 °C over the incubation period of 36 h (as opposed to the normal growth condition of 37 °C overnight) which does not rule out the possibility of non-specific complementation. It is important to note that both the strains JLD2402 and JLD2404 lack an overexpressing T7 polymerase gene, yet the pET15b-*FtglpX* vector could successfully complement both strains. This indicates a possibility of read-through from other promoters.

Since this complementation result is not confirmatory and rather non-specific, we investigated the possibility of pET15b-*FtglpX* vector complementing the JB108 strain of *E. coli*. The JB108 strain lacks the functional FBPase I (BL21(DE3) zjg920::Tn10 Δfbp287). JB108 is derived from the strain BL21(DE3) which has a host T7 polymerase gene that permits overexpression of a protein encoded in a pET vector [17]. *F. tularensis* FBPaseII protein expression from the T7 promoter complements growth of JB108 Δ*fbp E. coli* strain on glycerol minimal media in an IPTG concentration-dependent manner (Fig. 1b). Strains JB108, JB108::pET15b, and JB108::pET15b-*FtglpX*, in which *Ft*FBPaseII is overexpressed from the T7 promoter, and control strain BL21(DE3) (*fbp* ^+^ *glpX* ^+^) were grown in minimal glycerol-containing media. Variable concentrations of IPTG (0 to 1000 μM) were used. In addition, the growth of JB108::pET15b-*FtglpX* and the corresponding FBPase activity was directly proportional to the effective IPTG concentration used for growth (Table 1). The coupled spectrophotometric assay was used to measure the FBPase activity in cell-free extracts. As expected, the *E. coli* JB108 host and JB108::pET15b had very low FBPase expression levels, while the mutant complemented with pET15b-*FtglpX* showed elevated levels of FBPase activity in an IPTG concentration-dependent manner. This IPTG concentration-dependent expression of the FBPase indicates that pET15b-*FtglpX* successfully complements the *E coli* strain JB108.

**Table 1:** FBPase activity in *E. coli* strains expressing *Ft*FBPaseII protein

Strain/construct	[IPTG] (mM)	n	FBPase specific activity (nmol min^−1^ mg^−1^)
BL21(DE3)	0	3	16.30 ± 1.22
JB108	0	3	0.33 ± 0.08
JB108::pET15b	0	3	0.09 ± 0.03
JB108::pET15b	1000	3	0.16 ± 0.07
JB108::pET15b-*FtglpX*	0	6	0.33 ± 0.09
JB108::pET15b-*FtglpX*	50	6	5.53 ± 1.13
JB108::pET15b-*FtglpX*	250	6	9.89 ± 1.75
JB108::pET15b-*FtglpX*	500	6	16.72 ± 1.25
JB108::pET15b-*FtglpX*	1000	6	21.46 ± 1.87

**Fig. 2:**
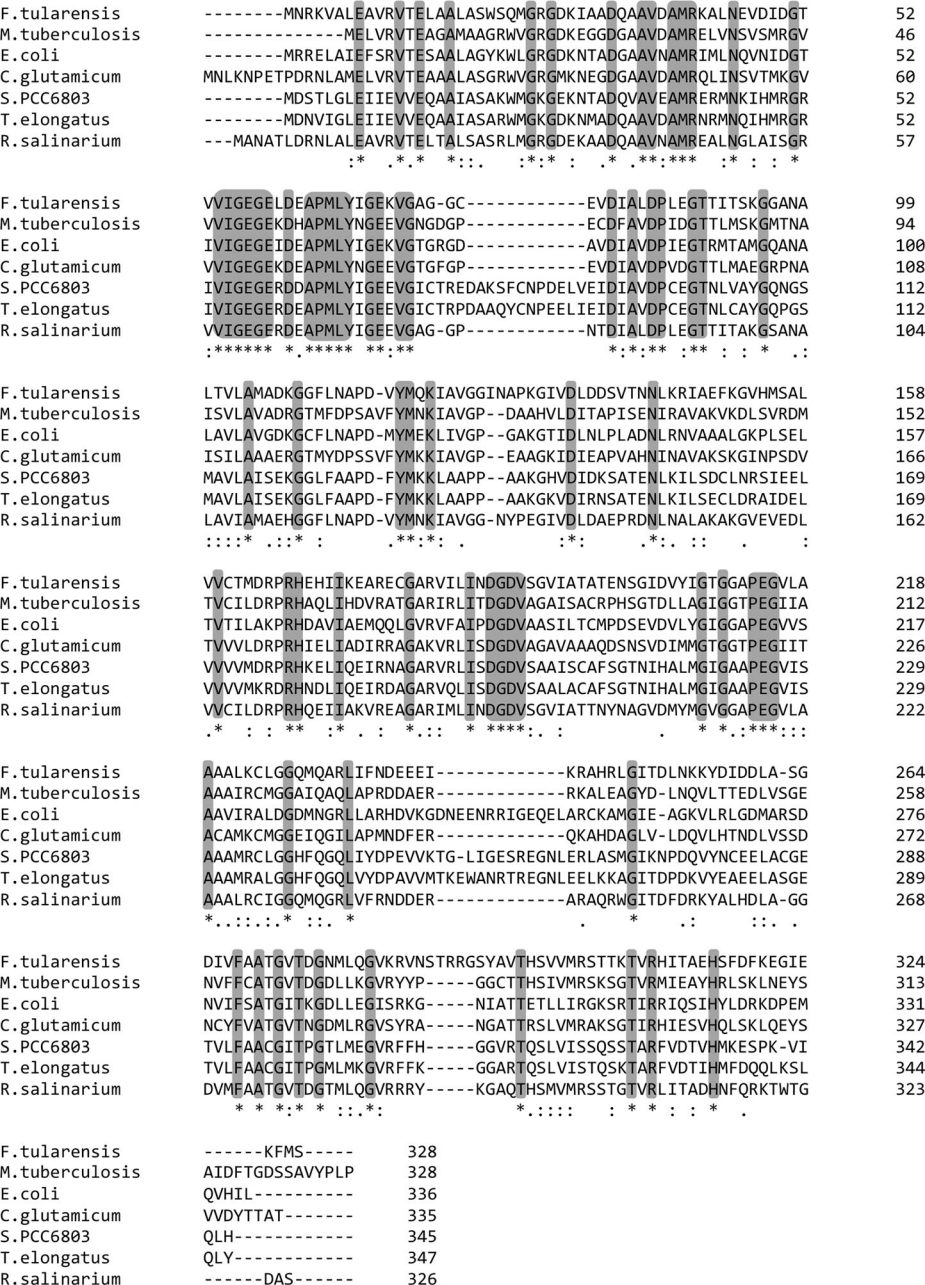
Sequence comparison of *FtglpX* gene with other class II FBPases. CLUSTALW alignment of *F. tularensis* FBPaseII with *M. tuberculosis (Rv1099c)*, *Escherichia coli* (PDB 3D1R), *Corynebacterium glutamicum* (Accession WP_003856830.1), *Synechocystis sp. PCC6803* (Accession WP_010872613), *Thermosynechococcus elongatus (dual FBPase/SBPase)* (PDB 5A5L), and *Rhodovibrio salinarium* (Accession WP_027288916.1) identified 73 conserved residues. The sequence identity ranged from 40.2 to 64.2% against *F. tularensis*. Invariant positions are indicated by *asterisks* (*) under the alignment, while highly conserved and weakly conserved positions are indicated by colons and periods, respectively

Primary sequence comparison of *Ft*FBPaseII, with other known class II FBPases (41–47% sequence identity), suggests that several regions are conserved. Furthermore, all the important catalytic residues are conserved (Fig. 2). Characteristic conserved regions have been indicated. Also, conserved residues adjacent to the catalytically important residues have been indicated.

### Purification

**Fig. 3:**
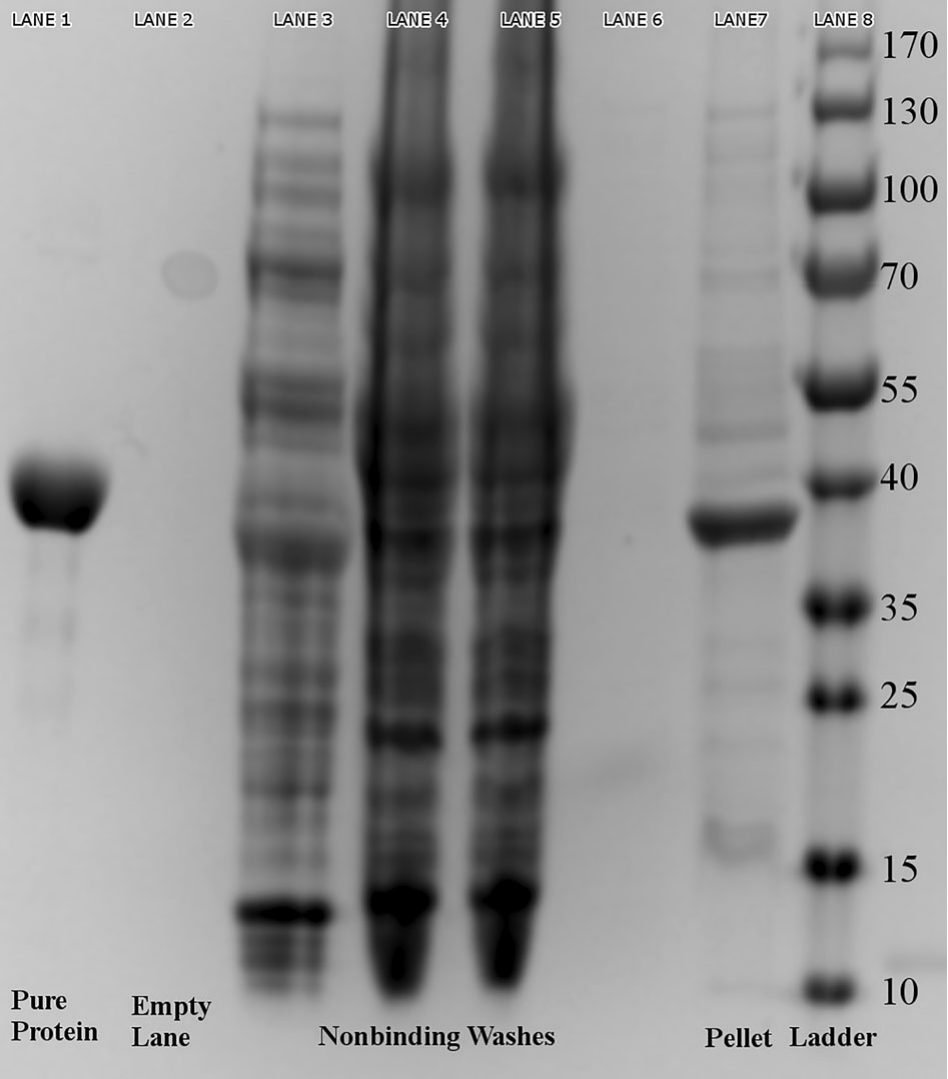
Purification of *Ft*FBPaseII. Purification of *Ft*FBPaseII was carried out with his-tag nickel affinity purification procedures, which resulted in highly pure protein of approximately 37 kDa, as seen in Lane 1. Lane 8 contains PageRuler Prestained Protein Ladder of indicated size bands in kDa. Lanes 3–6 contain non-binding washes. Lane 7 is the urea-soluble portion of the pellet

Significant expression of *Ft*FBPaseII was observed upon induction with 1 mM IPTG in the *E. coli* cell cultures transformed with the pET15b-*FtglpX* construct (not shown). The *Ft*FBPaseII, which was overexpressed in *E. coli*, was present in the soluble fraction and the insoluble fraction (Fig. 3, lane 7). The Ni-NTA elute showed no impurities in the gel (Fig. 3, lane 1).

**Fig. 4:**
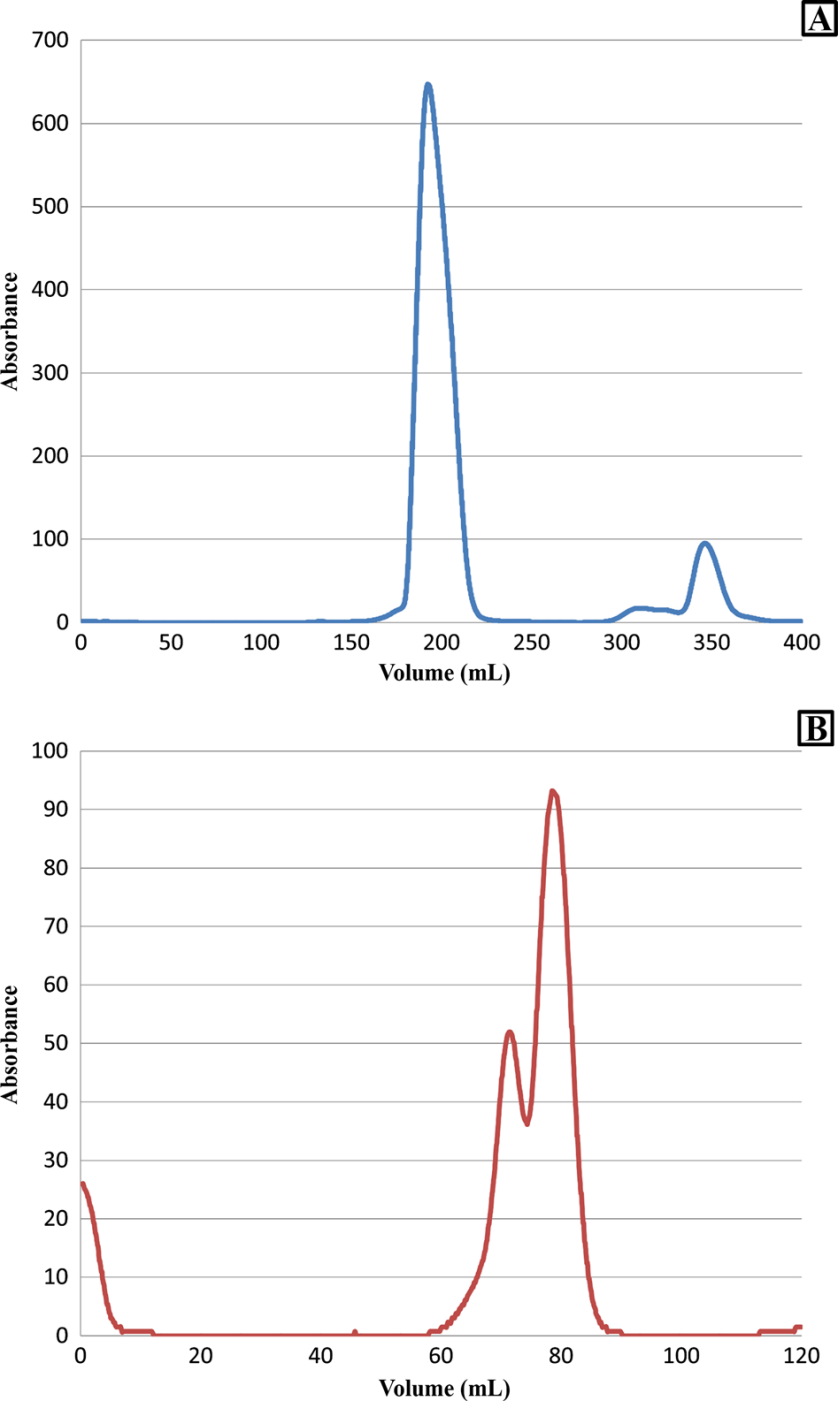
SEC chromatogram for *Ft*FBPaseII. A main peak at about 190 mL corresponds to the *Ft*FBPaseII tetramer (**a**). A small peak at 350 mL might be the diluted monomeric form of FBPase protein or other buffer ingredients. The peak maximum of first peak was about 650 mAu. SEC was repeated for *Ft*FBPaseII protein for molecular weight estimation (**b**). The chromatogram indicated the presence of dimeric and tetrameric species (63.8 and 124.9 kDa, respectively) by calculation from a calibration curve using molecular weight standards, thyroglobulin (bovine) 670 kDa, gamma globulin (bovine) 158 kDa, ovalbumin (chicken) 44 kDa, myoglobulin (horse) 17 kDa, and vitamin B12 1350 Da. Calibration curve can be found in previous publication [6]. Peak maximums are about 52 and 93 mAu

The protein was further purified with SEC. Fractions from the major peak at 200 mL appeared to have high purity as identified by SDS-PAGE (Fig. 4a) and, hence, were pooled. Since no protein was detected in the small peak at 350 mL by SDS-PAGE, it is assumed to contain dilute protein, non-protein impurities with A_280_ absorption, or other buffer ingredients. Protein concentration was determined by two methods. Comparison of the Pierce 660-nm kit and A280 with predicted extinction coefficient for protein quantification resulted in a ratio of the two predicted values to be 1.06 ± 0.10 (*n* = 3), confirming minimal variation between the two techniques.

### Molecular Weight Determination and Hydrodynamic Size

Mass spectrometry experiments indicated a mass of 36,846.6 g/mol, corresponding to the predicted size of 36,846 g/mol with the initial methionine cleaved in situ [18]. The oligomeric state of the *Ft*FBPaseII in solution state was evaluated by SEC. The void volume of the column, as determined by dextran blue, is *V*_*o*_ = 39.80 mL; total column volume, *V*_*t*_ (also referred to as geometric column volume) = 120 mL [6]. Using this method, two protein peaks corresponding to molecular weights of 63.8 and 124.9 kDa were observed (Fig. 4b). This observation suggests that at a concentration of 1 mg/mL, *F. tularensis* FBPaseII exists as a mixture of both dimers and tetramers, assuming the monomer subunit is about 35 kDa.

### Enzyme Activity

**Fig. 5:**
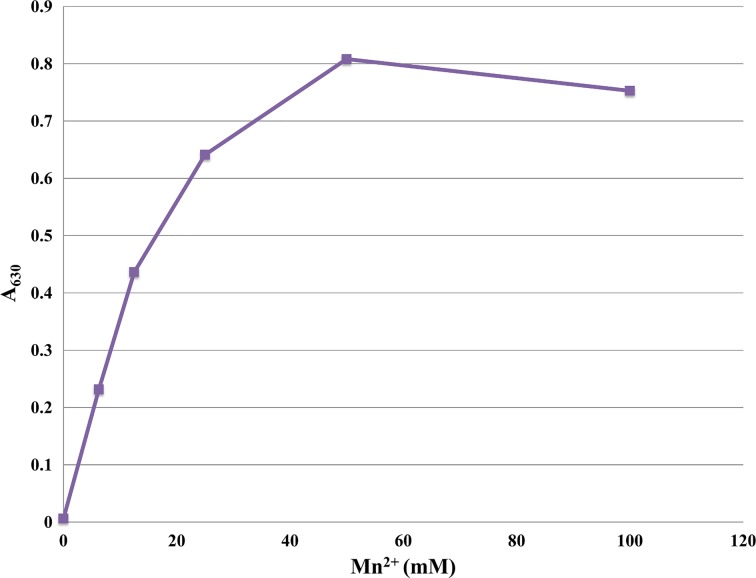
*Ft*FBPaseII activity is dependent on Mn^2+^. The activity of *Ft*FPBaseII was measured with a malachite green assay using 630 nm as detection wavelength. No activity was detected without Mn^2+^ and maximum activity was achieved at 50 mM Mn^2+^

**Fig. 6:**
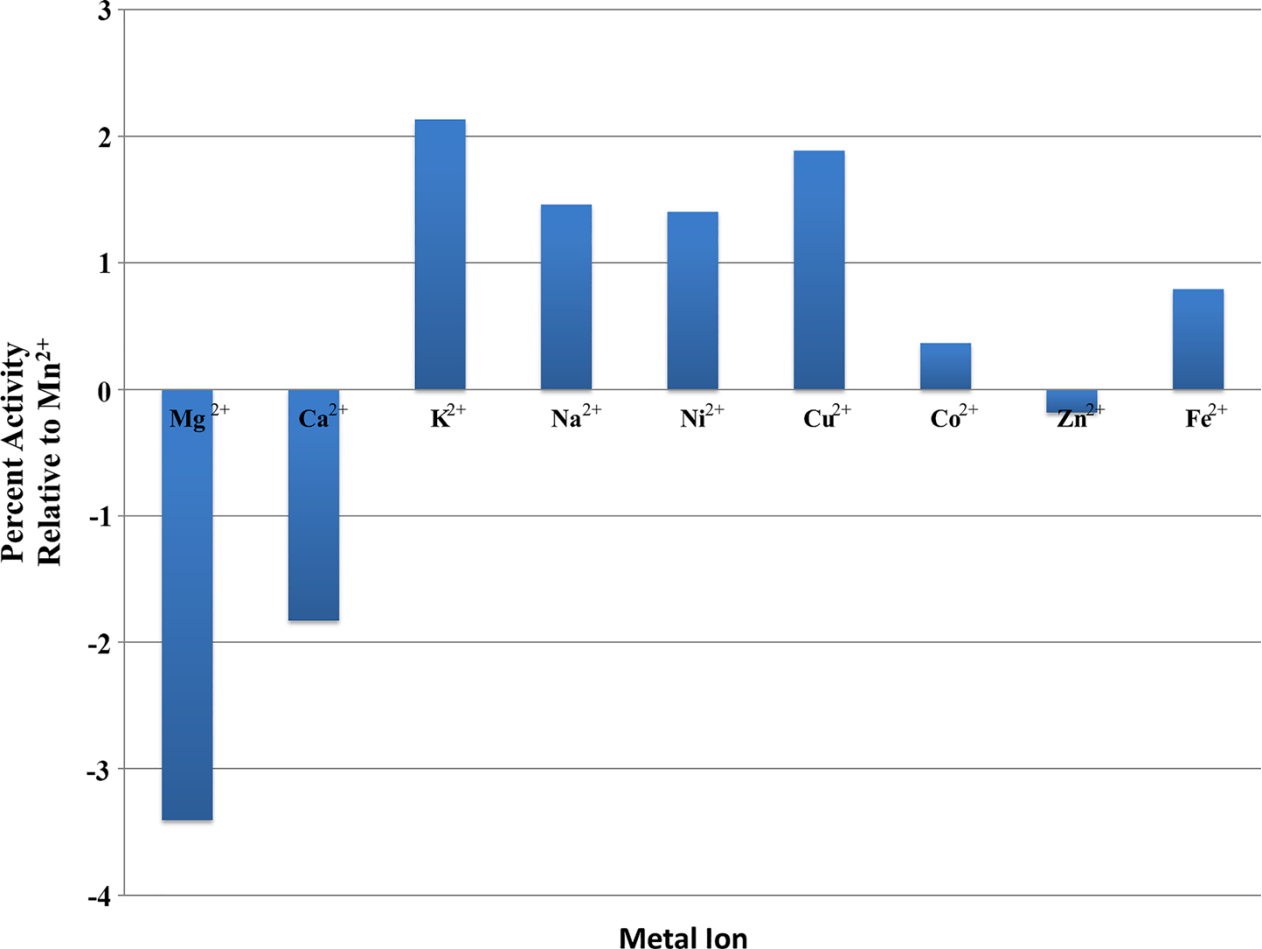
Metal requirement for *Ft*FBPaseII activity. Malachite green assay was conducted to find a metal ion that would allow for activity of *Ft*FBPaseII. Only the presence of Mn^2+^ in the assay allowed for sufficient production of inorganic phosphate to be detected

**Fig. 7:**
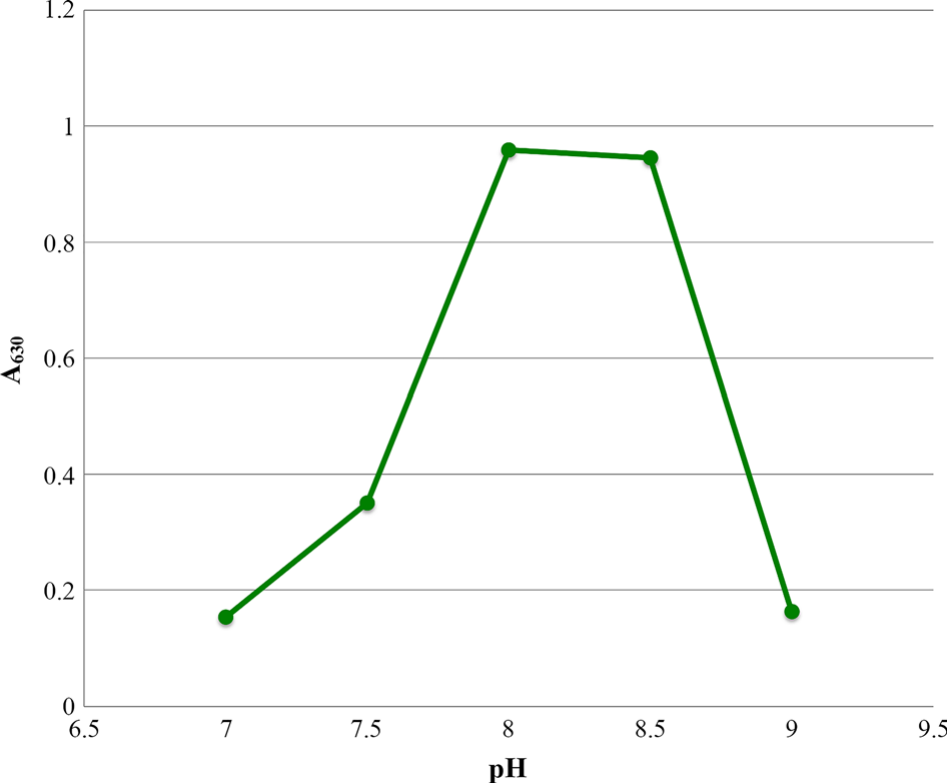
*Ft*FBPaseII activity is dependent on pH. Using a malachite green assay with 630 nm detection wavelength, the activity of *Ft*FPBaseII was optimal between 8.0 and 8.5 pH units in 50 mM tRIS buffer

**Fig. 8:**
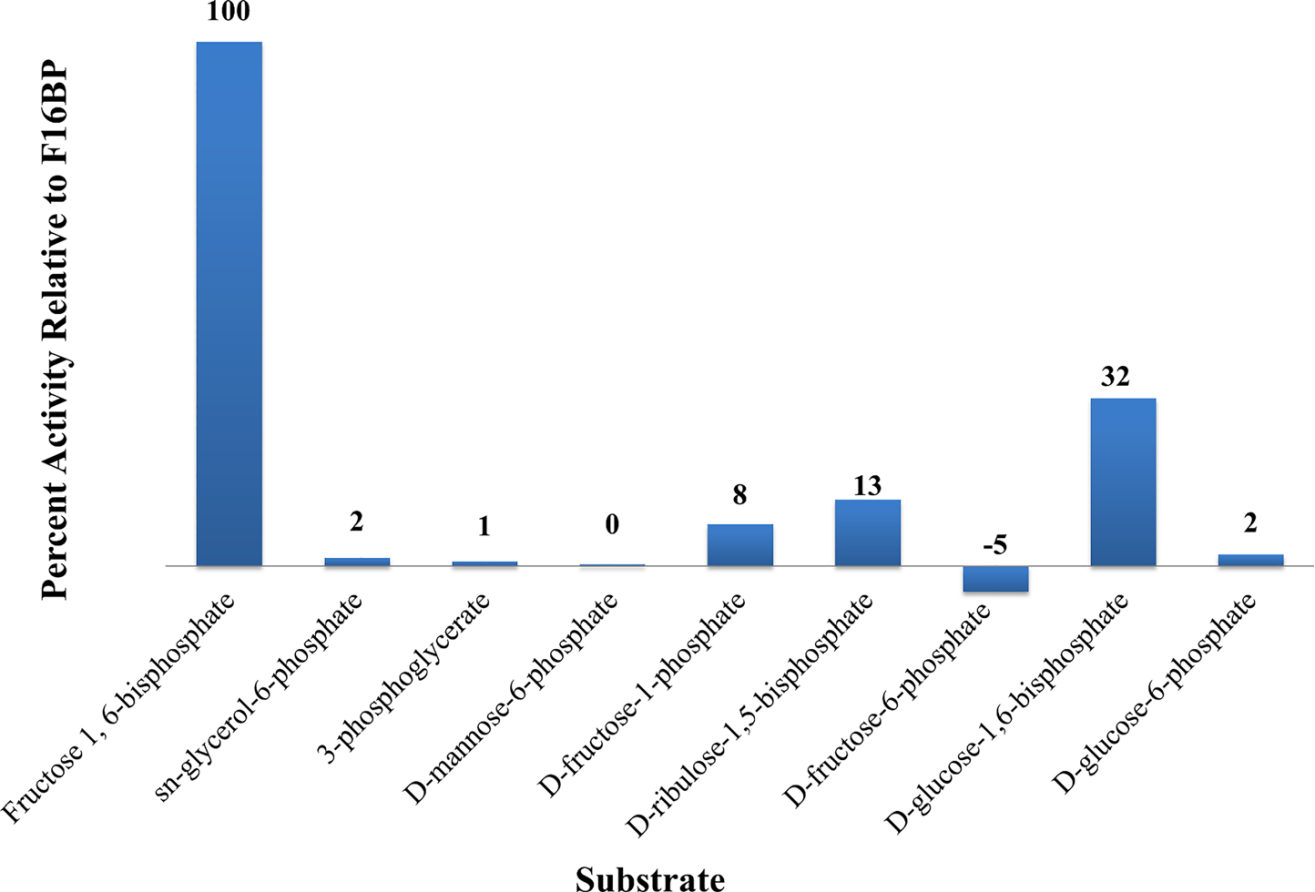
Substrate specificity for *Ft*FBPaseII activity. Malachite green assay was performed to quantify the production of inorganic phosphate from various substrates. F16BP had the greatest activity and is presumed to be the natural substrate. d-glucose-1,6-bisphosphate, d-ribulose-1,5-bisphosphae, and d-fructose-1-phosphate have limited phosphate liberation by *Ft*FBPaseII

**Fig. 9:**
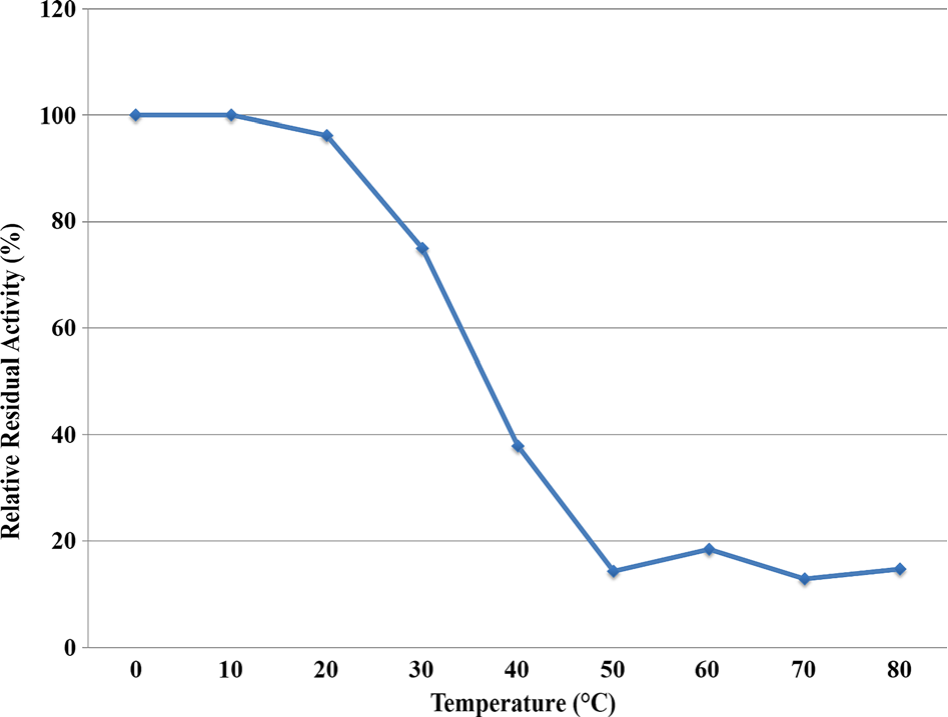
Relative residual activity of *Ft*FBPaseII after heating. Using a coupled assay, the activity of *Ft*FPBaseII was measured after 30 min at indicated temperatures. Activity is plotted relative to a sample incubated on ice. The protein remained stable when incubated below room temperature. At 30–40 °C, there was a marked decline in activity, and at 50 °C and above, activity of the enzyme was negligible

The activity of *Ft*FBPaseII was tested under various conditions using the malachite green assay. The bivalent metal, Mn^2+^, was found to be an absolute requirement for activity, with maximum activity at 50 mM Mn^2+^ (Fig. 5). None of the other bivalent or monovalent metals tested with *Ft*FBPaseII resulted in an active enzyme (Fig. 6). Activity of *Ft*FBPaseII was greatly reduced in tricine; slightly reduced in bis-tris; and showed similar levels of activity in tris, HEPES, and MOPS buffering systems. A buffer pH of 8.0–8.5 was optimal (Fig. 7). After a 10-min reaction of *Ft*FBPaseII with F16BP, 26 μM phosphate was released, which is a 26% turnover of substrate. As substrates, sn-glycerol-6-phosphate, 3-phosphoglycerate, d-mannose-6-phosphate, d-fructose-6-phosphate, and d-glucose-6-phosphate had less than 2% activity with *Ft*FBPaseII as compared to F16BP and hence are not substrates for the enzyme (Fig. 8). d-fructose-1-phosphate, d-ribulose-1,5-bisphosphate, and d-glucose-1,6-bisphosphate showed varying phosphate liberation by *Ft*FBPaseII with 8, 13, and 32% activity, respectively, when compared to activity with F16BP. The residual enzyme activity after heating experiments indicate highest activity with protein incubated at 0–20 °C; at 30 °C and above, the activity was reduced (Fig. 9).

Using a coupled assay with real-time measurements, K_m_ was found to be 11 μM, V_max_ was 2.0 units/mg, k_cat_ was 1.2 s^−1^, k_cat_/K_m_ was 120 mM^−1^ s^−1^, and specific activity of the enzyme was 2.0 units/mg. The R^2^ coefficient of determination was greater than 0.99. The reaction was linear for enzyme concentration up to 50 nM. *Ft*FBPaseII was inhibited by Li^+^ with an IC_50_ value of 100 mM in the coupled assay. Adenosine diphosphate had 28% inhibitory activity of *Ft*FBPaseII at 1 mM and precipitated at higher concentrations. High concentrations of phosphate also precipitated in the enzyme solution; nevertheless, 69% inhibition of the enzyme was detected at 150 μM.

## Discussion

Recent experiments have suggested the essentiality of the *glpX* gene in *F. tularensis* [3]. These studies followed the growth of *F. novicida*, a subspecies of *F. tularensis*, in glucose and glycerol. While these studies demonstrate that the *glpX*-encoded protein is required for growth on gluconeogenic substrates, they do not functionally verify the enzymatic activity of the encoded protein.

Our experimental plan to verify the functional activity was based on complementation of known *E. coli* mutants lacking significant FBPase activity by the *FtglpX*-encoded protein. While the complementation with *E. coli* strains JLD2402 and JLD2404 does not rule out non-specific complementation, it does indicate that the *FtglpX*-encoded protein helps such strains grow on gluconeogenic substrates.

Complementation studies show that the pET15b-*glpX* plasmid was able to restore growth of the *E. coli* strains (lacking a functional FBPase I) on glycerol and independently verify that the *glpX*-encoded FBPase of *F. tularensis* is a functional FBPase. Since the *E. coli* control strains do not have FBPase enzymes, it can be interpreted that FBPase activity is needed for growth on glycerol. This work independently verifies that the *glpX*-gene encoded protein in *F. tularensis* is indeed an FBPase which can successfully complement a Δ*fbp E. coli* strain (JB 108). Furthermore, the IPTG-induced overexpression of FBPase II from *F. tularensis* in a Δ*fbp E. coli* strain (JB 108) by pET15b-*FtglpX* proves that overexpression of this protein allows growth and proliferation of the *E. coli* strain.

Additionally, the primary sequence comparison of *Ft*FBPaseII with other known Class II FBPases also indicates that several regions are conserved and important catalytic residues in the enzyme are conserved. This work together with the complementation studies verifies that the encoded protein is an FBPaseII.

FBPase enzymes have been the subject of many drug discovery programs and other fields of research culminating with over 162 structures available in the protein data bank. A few of these enzymes have been subjected to extensive biochemical characterization, most notably, those from *E. coli* [14, 19], *Corynebacterium glutamicum* [15], and *M. tuberculosis* [6].

The *E. coli* FBPase is a dimer [14, 19] in solution while those from *C. glutamicum* [15] and *M. tuberculosis* [6] are both tetramers. Interestingly, we found that the quaternary structure was dependent on the protein concentration. While it is possible that there were two different molecular weight structures found at lower protein concentration, it is also possible that the protein had adopted two different conformations [20, 21]. In either case, we can conclude that the protein is more stable at the higher concentration.

*Ft*FBPaseII is dependent on Mn^2+^ for activity, as is the *E. coli* enzyme [14] while those from *C. glutamicum* and *M. tuberculosis* can use either Mn^2+^ or Mg^2+^ [15]. FBPase from *M. jannaschii* could substitute Zn^2+^ [13]. *F. tularenesis* was found to have weak sensitivity to Li^+^, similar to *E. coli* with an IC_50_ of 70 mM [19]. Li^+^ sensitivity in other organisms was more pronounced with IC_50_ values of 200 μM (*M. tuberculosis*) [6] and 140 μM (*C. glutamicum*) [15].

Substrate affinity and specificity between differing species’ enzymes may give us critical insight into active site differences. The K_m_ for *F. tularensis* is comparable to *C. glutamicum* with a K_m_ of 14 μM [15], while *E. coli* and *M. tuberculosis* K_m_ values are higher at 35 μM [14] and 44 μM [6], respectively. V_max_ of *C. glutamicum* of 5.4 units/mg [15] was highest, with *E. coli* Class II at 3.3 units/mg [13] and *M. tuberculosis* at 1.6 units/mg [6]. k_cat_ values of 1.0 [6] *M. tuberculosis* and 3.2 [15] *C. glutamicum* and 14.6 [22] *E. coli* Class I s^−1^. While k_cat_/K_m_ values had a larger range of 22.7 [6] (*M. tuberculosis*), 57 [14] (*E. coli* Class II), 948 [22] (*E. coli* Class I), and 236 mM^−1^ s^−1^ [15] (*C. glutamicum*). *E. coli* had low activity with substrates fructose l-phosphate and ribulose 1,5-bisphosphate [14] and glucose 1,6-bisphosphate [19]. *C. glutamicum* has low activity with glucose-6-phosphate [15].

FBPases are known to require bivalent metal ions and have sensitivity to lithium. These differences in absolute metal requirement, substrate affinity, substrate specificity, and lithium sensitivity may be the key indicators for targeting a particular enzyme for structure-based drug design [23]. The subtle active site changes from one species to another elucidated by structural analysis will give us critical insight. Substrate specificity differences give important enzymatic activity information for rational drug design [24]. With the aid of computational energy minimization, examination of the binding differences of the tested substrates could be used as a starting point.

It is assumed that inhibition of the enzyme by phosphate is due to binding of phosphate in the active site where the substrate’s own phosphate groups would bind. However, inhibition by adenosine diphosphate (ADP) brings up questions of the presence of an allosteric site. AMP is an allosteric inhibitor of human FBPase [25]. Future work to find the X-ray structure and further biochemical characterization with *Ft*FBPase and ADP should tell us the mechanism of inhibition.

The genetic complementation results prove that the *F. tularensis glpX* gene encodes for a protein that possesses FBPase activity and can complement the *E. coli ∆fbp* strain. Bioinformatics results indicate that it is a Class II FBPase. *Ft*FBPaseII was easily purified following standardized protocols. Biochemical characterization has provided valuable and novel information for drug discovery and should be pursued as an ongoing research activity.

The major bottlenecks in the process of structure-based drug discovery against this target are the availability of a purified protein target and the ability to crystallize the target in a robust crystal form. We have succeeded in the purification and biochemical characterization of the enzyme. The biochemical and structural understanding of this validated enzyme target can serve as a starting point for a structure-based drug discovery approach for this highly virulent bacterium.

## Abbreviations

F16BP: Fructose-1,6-bisphosphate
*fbp*: Class I Fructose-1,6-bisphosphatase gene
FBPaseII: Class II Fructose-1,6-bisphosphatase protein
*Ft*: *Francisella tularensis*
*Ft*FBPaseII: Fructose-1,6-bisphosphatase from *Francisella tularensis*
*glpX*: Class II Fructose-1,6-bisphosphatase gene
IPTG: Isopropyl β-D-1-thioglactopyranoside
JB108: *E. coli* BL21(DE3) strain lacking *fbp* gene
JB108::pET15b: JB108 transformed with empty plasmid
JB108::pET15b-*FtglpX*: JB108 transformed with plasmid containing *glpX* gene
JLD2402: *E. coli* strain lacking *fbp* gene and *glpX* gene
JLD2404: *E. coli* strain lacking *fbp* gene
SEC: Size exclusion chromatography
Tricine: N-(2-Hydroxy-1,1-bis(hydroxymethyl)ethyl)glycine
Tris: Tris(hydroxymethyl)aminomethane
HEPES: 2-[4-(2-hydroxyethyl)piperazin-1-yl]ethanesulfonic acid
MOPS: 3-Morpholinopropane-1-sulfonic acid
Bis-tris: 2-[Bis(2-hydroxyethyl)amino]-2-(hydroxymethyl)propane-1,3-diol
